
JOURNAL INFORMATION
==============================
NLM Title Abbreviation: Innov Surg Sci
Journal ID: Innov Surg Sci
NLM Journal ID: 101708165
Journal ID: iss

Innovative Surgical Sciences

EISSN: 2364-7485
Publisher: De Gruyter

ARTICLE INFORMATION
==============================
PMCID: PMC11917185
PMID: 40110107
DOI: 10.1515/iss-2025-2006
Article ID: iss-2025-2006
Article version: 1
Subjects: Article

Abstracts DGG

Electronic publication date: 2025 Mar 19
Publication date: 2025 Mar
Volume: 10
Issue: Suppl1
First page: 368
Last page: 370
Copyright: © 2025 by Walter de Gruyter Berlin/Boston
Copyright year: 2025
Copyright holder: De Gruyter
License: This work is licensed under the Creative Commons Attribution 4.0 International License.
License URL: https://creativecommons.org/licenses/by/4.0/

Page count: 3

==============================

Abstracts – DCK 2025 – 142nd Congress of the German Society of Surgery – Munich, March 26–28 • DOI 10.1515/iss-2025-2006
Innov Surg Sci 2025; 10 (Special Suppl 1): s368–s370

### Endovascular Treatment for Intact Splenic Artery Aneurysm is safe and effective

ID: 42

**Kategorie: DGG - Patientensicherheit in der Gefäßchirurgie**

Tina Cohnert[1 2], Gregor Siegl[1], Nicole Föger[1], Anneliese Baumann[1], Horst Portugaller[3]

[1]*Klin. Abt. f. Gefäßchirurgie, Medizinische Universität Graz, Graz, Österreich*, [2]*Gefässchir., Graz, Österreich*, [3]*Dept. of Neurointerventional, Vascular and Interventional Radiology, Graz Medical University, Graz*

**Background:**
Splenic Artery aneurysm is a rare intraabdominal aneurysm with a high risk of rupture in women during pregnancy. The optimal treatment for splenic artery aneurysm (SPA) is under discussion. In more centers endovascular treatment is applied (1), however the feasibility of this method is depending on the location of the SPA and ist proximity tot he splenic hilus. The treatment results for endovascular aneurysm exclusion and the necessity for secondary open vascular surgery were assessed.

**Materials and methods:**
Prospectively collected data on all patients who underwent splenic artery aneurysm repair between July 2005 and July 2024 were assessed retrospectively. Clinical data, CT angiography, MR angiography and Digital Substraction Angiography Images were analyzed. The long-term follow-up was completed. Pseudoaneurysms were excluded from this series.

**Results:**
A total of 34 patients (pts) underwent open surgical or endovascular treatment for asymptomatic SPA (10 men, 24 women, age 32-79 years, mean age 55.7±13.9 years).

In all patients the aneurysm was asymptomatic and incidentally detected during ultrasound or CT investigations. Aneurysm diamter at treatment was 1.5-7.0 cm (mean diameter 3.1±1.5 cm).

24 patients underwent primary endovascular treatment (coils n=16, stentgraft n=2, flow diverter stent n=2, combination n=4). Open vascular surgery was primarily performed in 10 patients. 8 patients underwent aneurysm resection and direct anastomosis of the splenic artery, 2 pts primary aneurysm resection in combination with splenectomy. All patients could be discharged home, without periinterventional complications.

During a mean follow-up of 72.8±64.2 months (range 1-221 months, median 60 months), 26 pts are alive, 4 lost to follow-up, and 4 deceased (2 cancer, 1 ruptured intacranial aneurysm, 1 old age). Secondary surgery for enlarging aneursms was required in 2 patients. Secondary endovascular intervention was performed in 3 patients, up to 4 re-interventions.

**Conclusion:**
Endovascular treatment of intact splenic artery aneurysms is safe and effective. Optimal diagnostic imaging is relevant for the development of an individual therapeutic strategy to ensure complete aneurysm exclusion and possibly splenic perfusion depending on localisation of the aneurysm and the degree of tortuosity of the splenic artery.

Open Access. © 2025 The Author(s), published by De Gruyter. CC BY This work is licensed under the Creative Commons Attribution 4.0 International License.

### Ten Year Time Trends of Amputation Surgery in Peripheral Arterial Disease in Germany: Before and During the COVID-19 Pandemic

ID: 70

**Kategorie: DGG - Patientensicherheit in der Gefäßchirurgie**

Konstantin Lukas Uttinger[1], Armin Wiegering[1], Sabine Steiner[2], Daniela Branzan[3]

[1]*Universitätsklinikum Frankfurt , Klinik für Allgemein, Viszeral- und Transplantationschirurgie, Frankfurt am Main, Deutschland*, [2]*Universitätsklinikum Leipzig, Abteilung für Angiologie, Leipzig, Deutschland*, [3]*Technische Universität München, Klinik und Poliklinik für Gefäßchirurgie , München, Deutschland*

**Background:**
Peripheral arterial disease (PAD) has been associated with suboptimal treatment, high mortality, and high amputation rates. It is unclear how the COVID-19 (coronavirus disease 2019) pandemic affected this development in a long term context.

**Materials and methods:**
This was a register based, retrospective, nationwide cohort study including patients hospitalised with PAD as a main or secondary diagnosis and amputation surgery between 2012 – 2021 in Germany. Primary endpoints were population wide major and minor amputation rates, in hospital mortality, and in hospital mortality rates. Secondary endpoints were same admission revascularisations and in hospital mortality in case of complications, i.e., failure to rescue (FTR). Pre-pandemic and pandemic trends, focusing on lockdown periods, were analysed.

**Results:**
A total of 365 926 patient records with PAD and amputation surgery were analysed. Median patient age was 75 years and 28.8% were female. Overall population wide amputation and in hospital mortality rates (monthly decrease – 0.002/100 000, p < .001, and –0.001/100 000, p < .001, respectively) and in hospital mortality rate (8.0% for 2012 – 2014 vs. 6.5% for 2020 – 2021; p < .001) declined between 2012 and 2020. Concurrently, same admission revascularisations increased (41.0% for 2012 – 2014 vs. 47.0% for 2020 – 2021; p < .001), while FTR decreased in a subset of complications (acute ischemia, major bleeding, compartment syndrome, and mesenterial ischemia). In the first pandemic lockdown, there was a temporary trend change to higher major amputations rates (0.02/100 000; p < .001) and higher in hospital mortality rates (+0.007/100 000; p <.001), which changed to a decrease as of the second lockdown (–0.03/100 000, p =.034, and –0.010/100 000, p < .001, respectively) in an interrupted time series analysis. There was no statistically significant change in observed amputation rates during lockdowns, while observed in hospital mortality rates decreased by 12.0% in the first lockdown (0.22/100 000 vs. 0.25/100 000; p = .005) compared with reference periods of the two previous years.

**Conclusion:**
Between 2012 and 2021, pre-pandemic trends toward decreasing population wide overall amputation rates, fewer major amputations, more amputation related revascularisation procedures, and lower in hospital mortality were maintained despite a temporary trend to increased major amputations and in hospital mortality during the first COVID-19-related lockdown in Germany.

### Impact of Patch Type on Wound Healing and Long Term Outcome after Endarterectomy of the Common Femoral Artery

ID: 716

**Kategorie: DGG - Qualitätsindikatoren in der Gefäßchirurgie**

Anna Stockinger[1], Gregor Siegl[1], Peter Konstantiniuk[1], Tina Cohnert[1]

[1]*Medical University of Graz, Department of Surgery, Division of Vascular Surgery, Graz, Österreich*

**Background:**
Endarterectomy of the common femoral artery (CFA) typically involves patch closure to prevent narrowing from sutures. Bovine pericardium provides an alternative to autologous patches from the great saphenous vein (GSV) or occluded superficial femoral artery (SFA). This of the shelf product is expected to reduce operating time, avoids additional incisions as well as impaired distal wound healing, and preserves the vein for future revascularization. However, few studies have compared the long-term success of different patch materials. This study aims to investigate the impact of various patch materials on wound complications and the 5-year outcome.g

**Materials and methods:**
Patients with isolated CFA endarterectomy at the Department of Vascular Surgery, University Hospital of Graz, performed in 2016 and 2017 were included. For wound healing complications data from medical records was retrieved. To investigate the five-year-outcomes patients were invited for a follow-up investigation. After giving informed consent, sonography of the operated CFA was performed to evaluate diameter and grade of restenosis if present.

**Results:**
Of 159 operated arteries, 129 met the inclusion criteria of isolated CFA endarterectomy. Postoperative wound complications were evaluated for all 129 arteries, Two cases (1.6% of 129) had to undergo acute revision for bleeding, lymphatic fistulas were seen in 5.4% (7/129), wound infection was diagnosed in 4.7% (6/129), necrosis or dehiscence of the wound edge was seen in 10.9%, negative pressure wound dressing was used in 1.6% (2/12), neither secondary wound closure nor split-thickness skin grafting was used. For statistical evaluation wound complication was defined as combined endpoint of the events listed above. No significant association was found between wound complications and patch material and neither for many other factors. Significant correlations were observed for gender (27.8% of men but only 9.4% of women had wound complications, $p = 0.03$), and operated side (31.8% of right sided but only 14.3% of lift sided operations presented wound complications, $p = 0,02$), .

Eighteen patients underwent a 5-year sonographic follow-up. A significant difference in CFA diameter between patch materials was identified: the median diameter was 14 mm with autologous patches and 10 mm with bovine pericardium ($p = 0.001$). No significant restenosis was observed. Patients with peripheral artery disease (PAD) stage IV received fewer venous patches than those with PAD stages I-III.

**Conclusion:**
No significant difference in wound complications between autologous and bovine patches was found. This challenges the belief that autologous material results in fewer complications and may reduce the need for vein harvesting in patients with impaired wound healing. It is unclear, if the the larger diameter of CFA's with autologous patches exists instantly after the operation due to a higher stretchability or if autologous patches enlarge over time. This question needs further investigations.
